# Impact of matrix-assisted laser desorption/ionization time of flight mass spectrometric evaluation on the clinical outcomes of patients with bacteremia and fungemia in clinical settings lacking an antimicrobial stewardship program: a pre-post quasi experimental study

**DOI:** 10.1186/s12879-018-3299-y

**Published:** 2018-08-09

**Authors:** Yong Duk Jeon, Hye Seong, Dokyun Kim, Mi Young Ahn, In Young Jung, Su Jin Jeong, Jun Yong Choi, Young Goo Song, Dongeun Yong, Kyungwon Lee, June Myung Kim, Nam Su Ku

**Affiliations:** 1 Department of Internal Medicine, Severance Hospital, Yonsei University College of Medicine, 50-1 Yonsei-ro, Seodaemun-gu, Seoul, 120-752 Republic of Korea; 2 AIDS Research Institute, Yonsei University College of Medicine, Seoul, South Korea; 3 Department of Laboratory Medicine, Yonsei University College of Medicine, Seoul, South Korea

**Keywords:** Matrix-assisted laser desorption/ionization time-of-flight, Antimicrobial stewardship program, Bacteremia, Fungemia, Clinical outcome

## Abstract

**Backgrounds:**

Several studies have evaluated the impact of matrix-assisted laser desorption/ionization time-of-flight (MALDI-TOF) mass spectrometry (MS) combined with antimicrobial stewardship in patients with positive blood cultures; clinical outcomes improved. However, in many hospitals, antimicrobial stewardship is not available because of restricted medical resources. Thus, we investigated the impact of evaluation by MALDI-TOF MS on the clinical outcomes of patients with bacteremia and fungemia treated in a clinical setting lacking an antimicrobial stewardship program (ASP).

**Methods:**

We designed a pre–post quasi experimental study and retrospectively reviewed the medical records of patients aged > 18 years old with bacteremia and fungemia during two periods: October–December 2012 and October–December 2013. Conventional methods were used to detect microbial pathogens in 2012, and MALDI-TOF MS was employed in 2013. Clinical outcomes compared between periods were the time to pathogen identification, time to effective therapy, 30-day all-cause mortality, time to microbiological clearance, length of ICU stay, and rate of recurrence of the same bloodstream infection (BSI).

**Results:**

A total of 556 patients were enrolled; 302 patients in 2012, and 254 in 2013. The use of MALDI-TOF MS without an ASP reduced the time to pathogen identification (86.4 vs. 63.5 h, *P* < 0.001) but did not significantly reduce the time to effective therapy (27.4 vs. 23.2 h, *P* = 0.187). Also, none of the following differed significantly between the two periods: mortality (17.5 vs. 15.7%, *P* = 0.571), the time to microbiological clearance (3.6 vs. 3.7 days, *P* = 0.675), the length of ICU stay (16.8 vs. 14.7 days, *P* = 0.706), and the recurrence rate of the same BSI (5.0 vs. 2.8%, *P* = 0.183).

**Conclusions:**

The use of MALDI-TOF MS alone in a setting lacking an ASP did not afford clinical benefits. An ASP combined with MALDI-TOF MS is necessary to improve clinical outcomes.

## Background

Despite considerable advances in medical technology and antibiotics, bloodstream infections (BSIs) are still associated with high-level mortality and morbidity [1]. The early administration of effective antibiotics is associated with improved clinical outcomes in patients with severe sepsis [2–5]. Similarly, early pathogen detection combined with appropriate therapeutic intervention improves the time to effective antimicrobial therapy and good clinical outcomes [6].

Matrix-assisted laser desorption/ionization time-of-flight (MALDI-TOF) mass spectrometry (MS) identifies microorganisms rapidly and accurately [7]. In clinical settings, MALDI-TOF MS reduces the time to identification by more than 24 h compared to conventional methods [8, 9].

Along with improvements in medical technology, antimicrobial stewardship programs (ASPs) have been introduced to care for septic patients and to optimize the use of antimicrobial agents [10]. However, in many hospitals around the world, ASPs are not in place because of restricted medical resources [11].

Several studies have shown that MALDI-TOF MS combined with an ASP improved clinical outcomes in patients with positive blood cultures [12–18]. Huang et al. [13] reported that the use of MALDI-TOF MS combined with an ASP in patients with bacteremia and candidemia was associated with improved clinical outcomes, including the time to effective therapy (30.1 vs. 20.4 h, *P* = 0.021), 30-day all-cause mortality (20.3% vs. 12.7%, *P* = 0.021), and length of intensive care unit (ICU) stay (14.9 vs. 8.3 days, *P* = 0.014) in univariate analyses, and that ASP introduction was related to a nonsignificant trend toward reduced mortality (odds ratio, 0.55; *P* = 0.075) in multivariate analysis.

However, the clinical responses afforded by MALDI-TOF MS-based evaluations alone have not been investigated. Thus, we explored the impact of MALDI-TOF MS on clinical outcomes (time to pathogen identification, time to effective therapy, 30-day all-cause mortality, time to microbiological clearance, length of ICU stay, and rate of recurrence of the same BSI) in patients with bacteremia and fungemia treated in a setting lacking an ASP. Because previous studies that evaluated the clinical impacts of other rapid diagnostic tests in the absence of an ASP failed to document the desired clinical outcomes [19, 20], we hypothesized that the introduction of MALDI-TOF MS alone would not improve clinical outcomes.

## Methods

### Study population and design

This retrospective, single-center, pre-post quasi-experimental study was conducted at Severance Hospital, a 2000-bed tertiary care hospital in Seoul, Korea. The electronic medical records of patients aged > 18 years old with positive blood cultures during two periods (October–December 2012 and October–December 2013) were reviewed. The conventional method (employing the ATB 32 GN system) was used to identify microorganisms in 2012, and MALDI-TOF MS was used in 2013. No ASP was in place during either period. Patients admitted to the institution for < 2 days after blood culture were excluded. The clinical outcomes were compared between the two periods. Our Institutional Review Board and local Ethics Committee approved the study.

### Definitions

Community-acquired BSI was defined as a BSI that developed within 48 h of hospitalization, and a hospital-acquired BSI was defined as a BSI developing ≥48 h after hospitalization [21]. A polymicrobial BSI was defined as a BSI associated with more than one microorganism.

Patients with cultures positive for coagulase-negative *Staphylococcus* and normal skin flora were screened as contaminants [13]. Contamination was considered present if coagulase-negative *Staphylococcus* was detected in only one of multiple blood cultures from the same patient, except when patients were suspected to have been infected via central venous catheters or foreign devices. We excluded patients with positive blood cultures, defined as contaminants.

The clinical outcomes compared were the time to pathogen identification, time to effective therapy, 30-day all-cause mortality, time to microbiological clearance, length of ICU stay, and 30-day rate of recurrence of the same BSI. The time to effective therapy was defined as the time from blood collection for culture to the time of administration of the first antimicrobial agent to which the pathogen was susceptible in vitro [13]. The time to microbiological clearance was defined as the time from the first positive blood culture to the first negative blood culture.

### Species identification and antimicrobial susceptibility testing

The microorganisms were identified using the ATB 32 GN system (bioMérieux, Marcy l’Etoile, France) in conventional period. In MALDI-TOF period, blood culture bottles were subcultured to the appropriate media and incubated aerobically and anaerobically for 18 to 24 h. Bacteria were applied as a thin film onto the plate and allowed to dry at room temperature. Subsequently, 2 μl of MALDI matrix was applied onto the colony and allowed to dry before testing. Analysis was done using MALDI Biotyper (Bruker Daltonics, Bremen, Germany). According to the criteria proposed by the manufacturer, an identification was considered reliable at the species level when the score was x ≥ 2 and at the genus level when the score was 1.7 ≤ x < 2 [21]. Antimicrobial susceptibility tests were performed using the disk-diffusion method or a VITEK-2 N131 card (bioMerieux, Hazelwood, MO, USA). The results were interpreted based on the Clinical and Laboratory Standards Institute (CLSI) guidelines.

### Statistical analysis

A statistical analysis was performed using SPSS for Windows (ver. 20.0; SPSS Inc., Chicago, IL, USA). Patient baseline characteristics, microorganism distributions, and clinical outcomes were compared between the conventional evaluation and MALDI-TOF periods. Continuous variables were compared using Student’s *t*-test; categorical variables were compared with the aid of the chi-squared or Fisher’s exact test. A two-sided *P*-value < 0.05 was considered to reflect statistical significance.

A subgroup analysis of infectious disease (ID) intervention was performed to evaluate the impact of MALDI-TOF MS combined with such intervention. ID intervention was defined as admission to the ID division, or ID consultation, within 3 days after blood cultures were drawn. The clinical outcomes of the two evaluation periods were compared between the ID intervention subgroups.

## Results

**Fig. 1:**
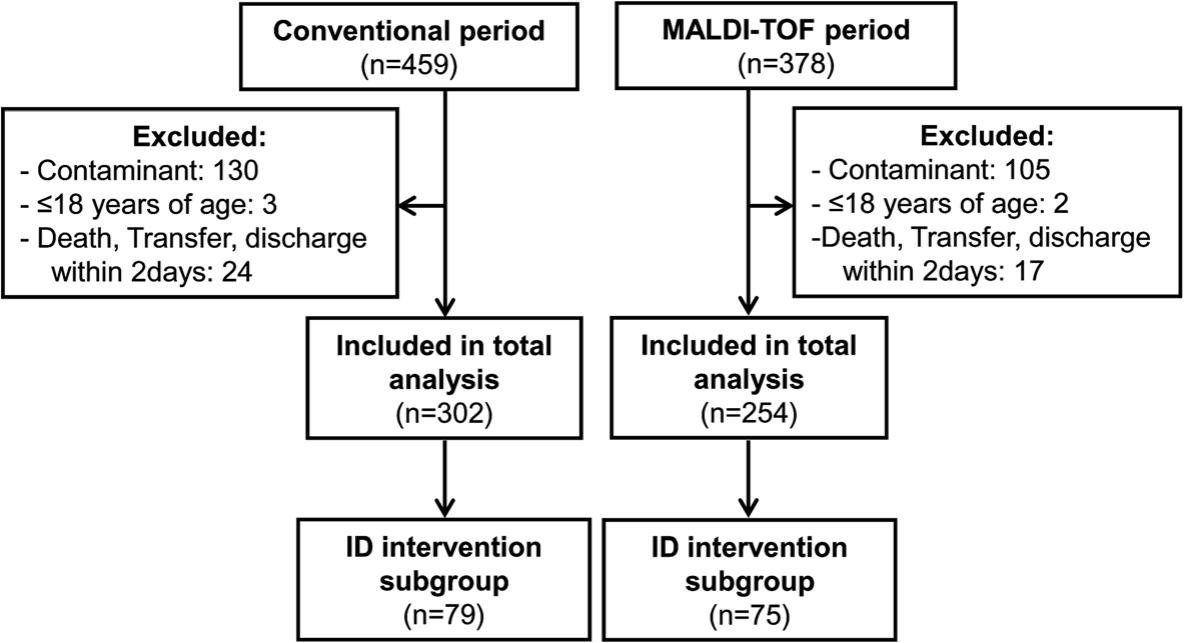
Flowchart of selected patients. *Abbreviations; MALDI-TOF, matrix-assisted laser desorption/ionization time-of-flight; ID, infectious disease

In total, 837 patients with positive blood cultures were identified during both study periods, and 556 were included in the final analysis (Fig. 1). The conventional period and MALDI-TOF MS period included 302 and 254 patients, respectively.

The baseline characteristics were similar between the two periods, except for the male: female ratio (Table 1). The patients in the MALDI-TOF group tended to be female (53.5 vs. 37.7% in the conventional period, *P* < 0.001), but no other demographic factor, including age, underlying disease, clinical condition, BSI acquisition (community- or hospital-acquired), or BSI source, differed significantly between the two periods. Sepsis-related organ failure assessment scores were calculated for all patients and did not differ significantly between the two periods (3.7 vs. 4.2, *P* = 0.110).

**Table 1:** Patient baseline characteristics

Baseline Characteristics	Conventional period *n* = 302 (%)	MALDI-TOF period *n* = 254 (%)	*P* Value†
Age (years), mean ± SD	63.5 ± 12.3	61.6 ± 13.9	0.088
Female	114 (37.7)	136 (53.5)	< 0.001
Underlying disease			
Solid tumor	169 (56.0)	133 (52.4)	0.396
Hematologic malignancy	27 (8.9)	20 (7.9)	0.653
Cardiovascular disease	54 (17.9)	54 (21.3)	0.316
Cerebrovascular disease	30 (9.9)	35 (13.8)	0.160
Chronic lung disease	7 (2.3)	9 (3.5)	0.389
Chronic liver disease	44 (14.6)	26 (10.2)	0.125
Chronic kidney disease	42 (13.9)	34 (13.4)	0.858
Transplantation	16 (5.3)	11 (4.3)	0.597
Charlson score, median (IQR)	6 (2–7)	6 (2–7)	0.505
Clinical conditions			
Prior Chemotherapy within 30 days	81 (26.8)	63 (24.8)	0.588
Neutropenia (ANC < 500 cells/μL)	26 (8.6)	19 (7.5)	0.627
ICU stay	51 (16.9)	47 (18.5)	0.618
SOFA score, mean ± SD	3.7 ± 3.6	4.2 ± 3.6	0.110
Acquisition of BSI			
Community acquired	190 (62.9)	165 (65.0)	0.617
Hospital acquired	112 (37.1)	89 (35.0)	0.617
Source of BSI			
Urinary tract	61 (20.2)	57 (22.4)	0.519
Respiratory tract	38 (12.6)	23 (9.1)	0.185
Biliary tract	73 (24.2)	63 (24.8)	0.863
Abdomen except biliary tract	29 (9.6)	23 (9.1)	0.825
Central venous catheter	32 (10.6)	27 (10.6)	0.990
Foreign device	5 (1.7)	2 (0.8)	0.462
SSTI/BJI	20 (6.6)	10 (3.9)	0.163
Infective endocarditis	6 (2.0)	4 (1.6)	0.761
Primary Unknown	38 (12.6)	45 (17.7)	0.091

*Abbreviations: MALDI-TOF matrix-assisted laser desorption/ionization time-of-flight, *IQR* interquartile range, *ANC* absolute neutrophil count, *ICU* intensive care unit, *SOFA score* sepsis related organ failure assessment score, *BSI* bloodstream infection, *SSTI* skin and soft tissue infection, *BJI* bone and joint infection

†*P*-values were calculated using the chi-square or Fisher’s exact test for categorical variables and Student’s *t*-test for continuous variables

The microorganism distributions were also generally similar between the two periods (Table 2). The polymicrobial BSI frequencies were 11.9% in the conventional period and 10.2% in the MALDI-TOF MS period (*P* = 0.530). The prevalence of Gram-positive bacteria (33.1 vs. 32.3%, *P* = 0.821), Gram-negative bacteria (61.5 vs. 60.4%, *P* = 0.762), and yeasts (5.3 vs. 7.4%, *P* = 0.294), did not differ significantly between the two periods.

**Table 2:** Microorganism distribution

Microorganism	Conventional period Number (%)	MALDI-TOF period Number (%)	*P* Value†
Polymicrobial BSI	36/302 (11.9)	26/254 (10.2)	0.530
Total number of organisms	338	285	
Gram-positive bacteria	112 (33.1)	92 (32.3)	0.821
*Staphylococcus aureus*	29 (8.6)	20 (7.0)	0.470
MRSA	16 (4.7)	12 (4.2)	0.754
Coagulase-negative *staphylococcus*	26 (7.7)	14 (4.9)	0.158
*Streptococcus* spp	16 (4.7)	23 (8.1)	0.087
*Enterococcus* spp	32 (9.5)	24 (8.4)	0.649
Other gram-positive bacteria	9 (2.7)	11 (3.9)	0.398
Gram-negative bacteria	208 (61.5)	172 (60.4)	0.762
*Escherichia coli*	94 (27.8)	81 (28.4)	0.866
*Klebsiella* spp	45 (13.3)	40 (14.0)	0.794
*Acinetobacter* spp	16 (4.7)	9 (3.2)	0.318
*Enterobacter* spp	12 (3.6)	5 (1.8)	0.170
*Pseudomonas aeruginosa*	6 (1.8)	6 (2.1)	0.765
*Citrobacter* spp	3 (0.9)	8 (2.8)	0.070
*Serratia* spp	3 (0.9)	3 (1.1)	> 0.999
*Proteus* spp	4 (1.2)	4 (1.4)	> 0.999
Other gram-negative bacteria	25 (7.4)	16 (5.6)	0.371
Yeast	18 (5.3)	21 (7.4)	0.294
Candida spp	17 (5.0)	21 (7.4)	0.224
*Cryptococcus* spp	1 (0.3)	0 (0.0)	> 0.999

*Abbreviations: *MALDI-TOF* matrix-assisted laser desorption/ionization time-of-flight, *BSI* bloodstream infection, MRSA methicillin resistant *Staphylococcus aureus*

†*P*-values were calculated using the chi-square or Fisher’s exact test

Compared to the conventional approach, MALDI-TOF MS performed in the absence of an ASP reduced the time to pathogen identification (86.4 vs. 63.5 h, *P* < 0.001) but did not significantly reduce the time to effective therapy (27.4 vs. 23.2 h, *P* = 0.187; Table 3), 30-day all-cause mortality (17.5% vs. 15.7%, *P* = 0.571), time to microbiological clearance (3.6 vs. 3.7 days, *P* = 0.675), length of ICU stay (16.8 vs. 14.7 days, *P* = 0.706), or 30-day rate of recurrence of the same BSI (5.0% vs. 2.8%, *P* = 0.183). The clinical outcomes did not differ significantly between the two periods.

**Table 3:** Clinical outcomes

Outcomes	Conventional period *n* = 302 (%)	MALDI-TOF period *n* = 254 (%)	*P* Value†
Time to pathogen identification (hours)	86.4 ± 30.0	63.5 ± 23.3	< 0.001
Time to effective therapy (hours)	27.4 ± 35.8	23.2 ± 34.2	0.187
30-day all-cause mortality	53 (17.5)	40 (15.7)	0.571
Time to microbiological clearance (days)	3.6 ± 2.4	3.7 ± 2.8	0.675
Length of ICU stay (days)	16.8 ± 31.7	14.7 ± 22.0	0.706
30-day Recurrence of same BSI	15 (5.0)	7 (2.8)	0.183

*Abbreviations: *MALDI-TOF* matrix-assisted laser desorption/ionization time-of-flight, *ICU* intensive care unit, *BSI* bloodstream infection

†*P*-values were calculated using the chi-square or Fisher’s exact test for categorical variables and Student’s *t*-test for continuous variables

Of the 556 study patients, 89 were hospitalized in the ID division and 65 were referred for ID consultation within 3 days after blood cultures were drawn. A total of 154 patients were thus included in the ID intervention subgroup; 79 and 75 in the conventional and MALDI-TOF MS periods, respectively. Subgroup analysis revealed that MALDI-TOF MS combined with the ID intervention significantly improved not only the time to pathogen identification (86.9 vs. 62.5 h, *P* < 0.001) but also the time to effective therapy (27.8 vs. 17.1 h, *P* = 0.031), compared to the conventional approach (Table 4). No other outcome, including 30-day all-cause mortality (8.9 vs. 6.7%, *P* = 0.612), the time to microbiological clearance (4.1 vs. 3.4 days, *P* = 0.081), the length of ICU stay (14.1 vs. 11.7 days, *P* = 0.719), and the 30-day recurrence rate of the same BSI (2.0 vs. 0.0%, *P* = 0.183), differed significantly during either period in the ID intervention subgroup.

**Table 4:** Clinical outcomes in Infectious disease Intervention subgroup

Outcomes	Conventional period *n* = 79 (%)	MALDI-TOF period *n* = 75 (%)	*P* Value†
Time to pathogen identification (hours)	86.9 ± 30.4	62.5 ± 23.1	< 0.001
Time to effective therapy (hours)	27.8 ± 32.4	17.1 ± 25.7	0.031
30-day all-cause mortality	7 (8.9)	5 (6.7)	0.612
Time to microbiological clearance (days)	4.1 ± 3.0	3.4 ± 2.0	0.081
Length of ICU stay (days)	14.1 ± 26.0	11.7 ± 12.8	0.719
30-day Recurrence of same BSI	2 (2.5)	0 (0.0)	0.497

*Abbreviations: *MALDI-TOF* matrix-assisted laser desorption/ionization time-of-flight, *ICU* intensive care unit, *BSI* bloodstream infection

†*P*-values were calculated using the chi-square or Fisher’s exact test for categorical variables and Student’s *t*-test for continuous variables

## Discussion

Most studies of the effects of MALDI-TOF mass spectrometric evaluation have been conducted in clinical settings featuring ASPs [12–18]. Of the hospitals included in one international survey, 58% had ASPs, but the rates were low in Africa (14%), South and Central America (46%), and Oceania (47%) [11]. Many hospitals do not adopt ASPs because of a lack of funds, personnel, and/or information technology, or prescriber opposition. However, few studies have explored the clinical outcomes afforded by MALDI-TOF MS in clinical settings lacking ASPs. In an observational study, Clerc et al. [21] explored the utility of the MALDI-TOF MS-based identification of Gram-negative pathogens in patients with BSIs who were not enrolled in an ASP, but included only patients who underwent ID consultations. We studied bacteremic and fungemic patients regardless of ID consultation status; this may reflect the real clinical situation in hospitals lacking ASPs.

We found that evaluation by MALDI-TOF MS in a setting lacking an ASP did not improve clinical outcomes. MALDI-TOF MS reduced the time to pathogen identification (86.4 vs. 63.5 h, *P* < 0.001), but the time to effective therapy did not significantly differ from that achieved with the conventional approach (23.2 vs. 27.4 h). Other outcomes, including 30-day all-cause mortality, the time to microbiological clearance, the length of ICU stay, and the 30-day recurrence rate of the same BSI, did not differ significantly between the two periods. Another study showed that MALDI-TOF MS used with an ASP reduced the time to pathogen identification (84 vs. 55.9 h, *P =* 0.001), leading to improved clinical outcomes [13]. These findings are consistent with those of previous studies evaluating the clinical impacts of other rapid diagnostic tests in the absence of ASPs [19, 20]. Trep et al. [19] evaluated the impact of rapid methicillin-resistant *Staphylococcus aureus* polymerase chain reaction testing in patients with purulent skin infections. Clinical outcomes did not improve; the introduction of a rapid diagnostic test in the absence of an effective implementation strategy failed to produce the desired results. Another study investigated the impact of peptide nucleic acid fluorescence in situ hybridization evaluation in patients with coagulase-negative *Staphylococcus* bacteremia [20]. Again, no clinical benefit in terms of the length of hospital stay or vancomycin use was apparent in the absence of an ASP.

The use of MALDI-TOF MS significantly reduced the time to effective therapy in the ID intervention subgroup; the time was 27.8 h in the conventional period, and 17.1 h in the MALDI-TOF MS period (*P* = 0.031). This finding suggests that the ID intervention helped to reduce the time to effective therapy, in line with the findings of other reports showing that MALDI-TOF MS combined with antimicrobial stewardship improves clinical outcomes [12–18]. Our findings thus support the importance of the role of the ID specialist and an ASP.

Huang et al. [13] reported that MALDI-TOF with ASP in patients with bacteremia and candidemia reduced not only time to effective therapy (30.1 vs 20.4 h, *P* = 0.021) but also 30-day all-cause mortality (20.3% vs 12.7%, *P* = 0.021) and length of ICU stay (14.9 vs 8.3 days, *P* = 0.014) in univariate analysis. In the present study, ID subgroup analysis revealed no significant reductions in 30-day all-cause mortality (8.9% vs. 6.7%, *P* = 0.612) or length of ICU stay (14.1 vs. 11.7 days, *P* = 0.719). However, the ID intervention of our study was not equivalent to an ASP. The principal focus of our study was on whether evaluation by MALDI-TOF MS in a setting lacking an ASP improved clinical outcomes; we found that it did not.

Our study has several limitations. First, this was a single-center retrospective cohort study. It may thus be difficult to generalize our results. Also, any retrospective study may be subject to selection or information bias. Second, we did not calculate the appropriate sample size, which may have contributed to the non-significance of the difference in time to effective therapy (27.4 vs. 23.2 h, *P* = 0.187) in the total group. Third, we censored data from patients who did not receive effective antibiotics with in vitro activity, but we used no other technique to handle censored data. Fourth, follow-up blood culture was not the same for all patients because this was a retrospective cohort study. Differences among clinicians in the approach to follow-up blood culture may have resulted in ascertainment bias.

## Conclusions

Our results suggest that the use of MALDI-TOF MS alone (thus, in a setting lacking an ASP) did not improve the clinical benefits afforded to patients with bacteremia and fungemia. Our study strengthens the suggestion that an ASP is important when seeking to enhance clinical outcomes.
